# Rewriting Approaches for Ontology-Mediated Query Answering

**DOI:** 10.1007/s13218-020-00671-w

**Published:** 2020-06-11

**Authors:** Shqiponja Ahmetaj

**Affiliations:** Institute of Logic and Computation, TU Wien, Vienna, Austria

## Abstract

A most promising approach to answering queries in ontology-based data access (OBDA) is through query rewriting. In this paper we present novel rewriting approaches for several extensions of OBDA. The goal is to understand their relative expressiveness and to pave the way for efficient query answering algorithms.

## Introduction

*Ontology-based data access* plays a key role in current information management systems, receiving tremendous attention over the past decade in the areas of Knowledge Representation (KR), Semantic Web and Databases. It aims to integrate incomplete and heterogeneous data sources and to enrich the query answers by means of an intermediate layer that conceptualizes the domain of interest, known as an *ontology*.

A crucial task of OBDA is to answer user queries over incomplete data by employing the implicit information provided by the ontology. This problem is known as *ontology-mediated query answering* (OMQA). In this setting, the query and the ontology are naturally viewed together as a composite query called *ontology-mediated query* (OMQ). The main languages for writing ontologies are *Description Logics* (DLs) [6], a family of decidable fragments of first-order logic with at most binary predicates, and $\textsf {Datalog} {\pm }$ [10], a family of related rule-based languages. DLs, in particular, play a pivotal role in the context of Semantic Web as they underlie the Web Ontology Language (OWL 2) and its profiles [14]. The typical query languages adopted in OMQs are *conjunctive queries* (CQs), which correspond to the *select-where-from* fragment of SQL.

The standard way of answering OMQs, is to compute the *certain answers*, which, intuitively, are those that are true in all the possible worlds that satisfy the database and the ontology. As a simple illustration of OMQA, following our example in [3], consider a database that stores that Emma is an undergraduate student and Alvjen is a student; in our case, written as a DL *ABox* with two facts $\{\mathsf {UnderGradStudent}(a),\mathsf {Student}(b)\}$. If we pose to the database the *atomic query* $\mathfrak {q} (x) = \mathsf {Student}(x)$, that asks to retrieve all students, then the only answer is *b* (Alvjen). Now, consider an ontology capturing the intuitive knowledge that undergraduate students are students; written in DL syntax as a so-called *TBox* $\mathcal {T}$ with one axiom:

1 $$\begin{aligned} \mathsf {UnderGradStudent} \mathbin {\sqsubseteq }\mathsf {Student} \end{aligned}$$

If we pose the OMQ $(\mathcal {T}, \mathfrak {q})$ over the database, we retrieve the expected answers, i.e. both *a* (Emma) and *b* (Alvjen).

**Fig. 1:**
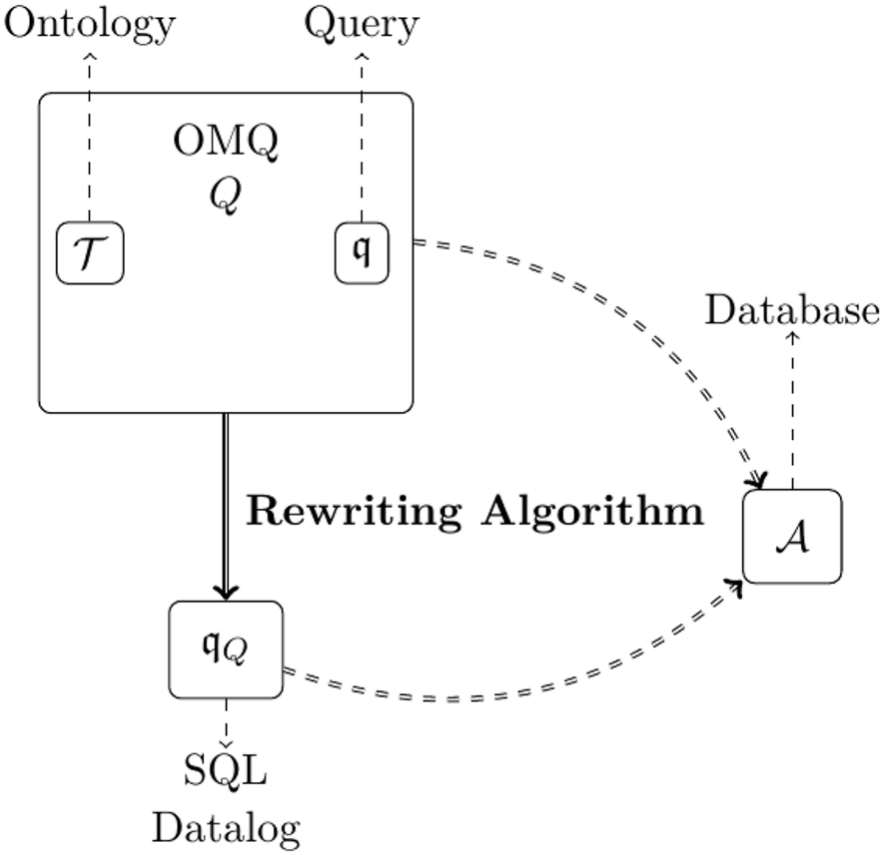
OMQA via rewriting

To make OMQA scale to increasing amounts of data, it is essential to exploit standard database systems. The latter have been optimized through decades of research and are very efficient in practice. Such systems, however, cannot support ontologies. A most promising solution is to *rewrite* the input OMQ into a new query, in a standard query language, which embeds the relevant knowledge implicit from the ontology; this query can then be answered directly over the data. This is referred to as *OMQ rewriting* and is considered to be a standard approach for OMQA. The underlying idea of OMQ rewriting, also depicted in Fig. 1, is as follows: an OMQ $Q =(\mathcal {T},\mathfrak {q})$, consisting of an ontology $\mathcal {T}$ and a query $\mathfrak {q}$, is rewritten into a new query $\mathfrak {q} _Q$, also called a rewriting, that provides the same certain answers as *Q* over all input databases.

Consider again the OMQ $(\mathcal {T},\mathfrak {q})$. A rewriting of $(\mathcal {T},\mathfrak {q})$ is the new query $\mathfrak {q} '(x)$, which results from $\mathfrak {q} (x)$ by replacing $\mathsf {Student}(x)$ with $\mathsf {Student}(x) \vee \mathsf {UnderGradStudent}(x)$ We can now pose to the database alone the query $\mathfrak {q} '$, which asks for all those that are students or undergraduate students, and we obtain both *a* and *b* as answers. Intuitively, this is because $\mathfrak {q} '$ captures the two possible ways to infer from $\mathcal {T}$ that someone is a student.

The most desirable target language is the class of *first-order (FO) queries*, (or, equivalently, (nonrecursive) SQL queries) since it enables the exploitation of SQL engines, which are very efficient in practice. However, this is often not possible even for simple OMQs. Consider the OMQ pairing a TBox with one axiom $\{\exists \mathsf {hasIngredient.Active} \mathbin {\sqsubseteq }\mathsf {Active}\}$, stating that a substance with an active ingredient is also active, with the atomic query $\mathsf {Active}(x)$. It is known that this OMQ cannot be rewritten as a FO query; see, e.g. [16]. Nevertheless, it can be expressed as the Datalog rule: $\mathsf {Active}(x) \leftarrow \mathsf {hasIngredient}(x,y) \wedge \mathsf {Active}(y)$. Note that, in general, a rewriting is not just a conversion of the axioms. It is thus crucial to understand what is the possible target language for different families of OMQs (*relative expressivenes*) and whether they admit succinct rewritings (*succincntess*). The ontology languages studied in our work can all express OMQs like the one above, which means that we cannot target FO queries, but rather more expressive languages, such as variants of Datalog.

Despite the fact that OMQA has been a subject of in-depth study, many challenges remain to be addressed. Notably, several extensions of the components of the OMQA framework, that enhance its expressive power and application domain, have been subject of active research. In the doctoral dissertation [1], we study three particular ways that enrich OMQA: (1) the database is viewed as partially complete through the so-called *closed predicates*, (2) the ontology languages are given in terms of *expressive* DLs and $\textsf {Datalog} {\pm }$ languages, and (3) the query language is given in terms of a *fragment of SPARQL*, the standard query language for the Semantic Web. The goal is to investigate OMQA in the presence of such extensions and to explore novel rewriting techniques, with the emphasis on succinct rewritings.

In this article, we present an overview of our results for the first two items. The aim is to better understand the relative expressiveness and succinctness of such OMQs, and thus to pave the way for efficient OMQA algorithms. In a somehow different spirit, we propose rewritings for OMQA in the context of Semantic Web (item 3 above), where we argue that the standard semantics, in a specific setting, often misses intuitive answers. For more details, we refer the reader to [2].

## Succinct Rewritings for Expressive Ontology Languages and Closed Predicates

*Mixing complete and incomplete information* To deal with the incompleteness of the data, OMQA usually employs the *open-world assumption* (OWA), that is no assumptions can be made on the facts not present in the data. In our example, Emma is a student, although this fact is not in the database. There are many scenarios, however, where viewing the data as partially complete is more suitable. Recent years have seen a renewed interest in this topic in the database and KR communities, e.g. when data coming from the web interacts with a relational store. In the context of DLs, this has been achieved through *closed predicates*, introduced in [19], which refer to a set of predicates that are assumed complete and have closed-world semantics [17, 18]. In particular, assuming some predicates as closed, may enrich the query answers. Following the example in [3], consider an extension $\mathcal {T}'$ of our $\mathcal {T}$ with two axioms, stating that every student attends some course and undergraduate students cannot attend graduate courses:

2 $$\begin{aligned} \mathsf {Student}&\mathbin {\sqsubseteq }\exists \mathsf {attends.Course}, \end{aligned}$$

3 $$\begin{aligned} \mathsf {UnderGradStudent}&\mathbin {\sqsubseteq }\forall {\mathsf {attends}}.{\lnot \mathsf {GradCourse}} , \end{aligned}$$

and consider the ABox $\mathcal {A}$ with assertions $\mathsf {Course}(c_1)$, $\mathsf {Course}(c_2)$, $\mathsf {GradCourse}(c_2)$, and $\mathsf {UnderGradStudent}(a)$. There are no certain answers for the OMQ $(\mathcal {T}',\mathfrak {q} ')$, where $\mathfrak {q} '(x,y)=\mathsf {attends}(x,y)$. Intuitively, we cannot infer which course Emma attends. Conversely, $(a,c_1)$ becomes a certain answer if we declare $\mathsf {Course}$ a closed predicate, i.e. we know $c_1$ and $c_2$ are the *only* courses. Note that Emma cannot attend $c_2$ because of axiom (3).

Interestingly, allowing closed predicates makes query answering *non-monotonic*, i.e., expanding the database may reduce the certain answers. Consider the new ABox $\mathcal {A}'$, which adds in $\mathcal {A}$ a new fact $\{\mathsf {Course}(c_3)\}$. Then, $(a,c_1)$ is not anymore a certain answer over $\mathcal {A}'$.

Most related works have focused on the complexity of query evaluation. There is very little work studying their expressiveness relative to the standard query languages. From the above observations, to capture the effect of closed predicates, it is crucial that the target query language is able to express non-monotonic behavior.

*Expressive ontology languages* $\textsf {Datalog} ^{\pm }$ languages (or classes of *tuple-generating dependencies* (TGDs)) capture and even generalize a wide variety of DLs. In particular, it has been widely accepted that it is convenient to use them for handling arbitrary arity relations, while DLs usually operate on predicates of arity at most two. For instance, the DL axiom (2) can be equivalently expressed as the following TGD $\forall x.(\mathsf {Student}(x) \rightarrow \exists y. \mathsf {attends}(x,y) \wedge \mathsf {Course}(y)).$

There is a wide variety of DLs and $\textsf {Datalog} ^{\pm }$ languages differing in the knowledge they can express. The vast majority of the works on rewritings are for OMQs whose ontological component is in the less expressive spectrum and use as target languages mainly FO queries, see e.g. [9, 12, 13]. Although such languages are shown to be useful for some practical scenarios, they are, however, not expressive enough to capture many real-life ontologies. For instance, a key and natural feature they cannot express is disjunctive information such as the axiom $\mathsf {Student} \mathbin {\sqsubseteq }\mathsf {UnderGradStudent} \sqcup \mathsf {GradStudent}$ stating that every student is undergraduate or graduate. Crucially, existing rewritings, e.g. [7, 8, 11, 15], are typically of exponential size, thus generating exponentially larger programs in variants of Datalog.

Polynomial-size (in polynomial time) rewritings are the most desired in the OMQA setting as they allow an efficient reduction of OMQA to (plain) database query evaluation. The existence of such succinct rewritings for different OMQ languages, and their computational cost have been considered a major research problem. For expressive ontology languages, particularly in the presence of disjunction, the existence of polynomial rewritings has been considered an open problem. We contribute to filling this gap by investigating novel approaches which generate succinct rewritings for rich ontology languages.

## Rewritability Results

We propose polynomial-size rewritings for ontology languages that support disjunction, with and without the presence of closed predicates. We note that, to the best of knowledge, no prior polynomial time rewritings existed for the classes of OMQs considered here.

To obtain such rewritings is a highly non-trivial task. Existing ones cannot be directly employed as they are all exponential. To design a rewriting into variants of Datalog, we need to overcome the substantial limitation that they cannot access the anonymous domain elements that ontologies can express through the existential quantification on the right-hand side of axioms (or TDGs), and to carefully encode all the features of the ontology while remaining in polynomial time. In addition to the expressiveness of the ontology language, the non-monotonicity caused by the presence of closed predicates makes finding such a suitable succinct rewriting even more challenging. Furthermore, the difficulty is amplified for $\textsf {Datalog} ^\pm$ ontology languages because of the higher arity predicates.

- In the context of DLs, we focus on the expressive DL $\mathcal {ALCHOI}$ [3, 4], which allows for conjunction, disjunction, existential and universal quantification, inverse roles, role hierarchies, and nominals (or constants). We propose a novel and versatile rewriting technique for OMQs, where the ontology is formulated in $\mathcal {ALCHOI}$ in the presence of closed predicates and the query is from a restricted class of CQs that generalizes the well-known acyclic CQs. The result is a polynomial time rewriting into Datalog with stable negation.
  The technique relies on a type-elimination style algorithm which essentially decides whether a given input structure can be extended to satisfy the input ontology while preserving the entailment of ground atoms. Roughly, the algorithm first builds all the possible *types*, where a type intuitively describes a node in a tree model; then it marks all the types that violate some axiom in the input ontology and for which no suitable successor type exists. If none of the types extracted from the input structure are marked, we conclude that it can be extended into a model. This intuition is nicely captured by a simple and intuitive game with two players, which we use to prove the correctness of the algorithm.
  For the DL $\mathcal {ALCHI}$, i.e. $\mathcal {ALCHOI}$ without nominals $\mathcal {O}$ and closed predicates, we obtain a polynomial time rewriting into positive Datalog with disjunction. We refer to [3, 4] for more details.
- In the context of $\textsf {Datalog} ^\pm$, we focus on guarded (disjunctive) TGDs [5], i.e. the class of TGDs that have an atom on the left-hand side that contains all the universally quantified variables. We have shown that, despite the rather relaxed syntax and high-arities of predicates, we can adapt our technique to support OMQs over *guarded disjunctive* TGDs and a class of CQs, similar to the one described above, to obtain a rewriting into Datalog with disjunction. The notion of a type, and consequently the game and the algorithm, become significantly more involved. The rewriting is polynomial if there is a bound on the arities of the predicates and number of variables. If no restrictions are imposed, the rewriting is exponential, which is unavoidable considering their complexities and the usual assumptions in complexity theory, i.e. the existence of a polynomial rewriting would imply co$\textsc {NExpTime} =\textsc {2ExpTime}$.
  As a final expressive ontology language, we study guarded (non-disjunctive) TGDs. The central result is a novel polynomial time rewriting of such OMQs into (non-disjunctive) Datalog programs if we assume the restrictions mentioned above; otherwise it is exponential. To obtain a polynomial translation into plain Datalog, we propose a novel algorithm which supplements our type-elimination style algorithm.
- In our rewritings, we make use of two distinct constants, which turn out to be essential to obtain a rewriting in polynomial time. Indeed, we show that if no constants are allowed in the rules, polynomial time rewritings into variants of Datalog for the above classes of OMQs do not exist.

## Conclusion

The goal was to develop novel and succinct rewriting approaches for the problem of OMQA in three particular extensions of the classical setting. Here, we presented the results for two of them. Crucially, the DL $\mathcal {ALCHOI}$ covers most of the popular constructs of the logic underpinning OWL 2 and guarded disjunctive TGDs capture and even generalize $\mathcal {ALCHI}$ by allowing for predicates of arbitrary arity. Our results show that although the OMQs we consider are very rich and permit extensive ontological reasoning, they can be efficiently rewritten (in polynomial time) as succinct Datalog programs with disjunction or stable negation. The latter are very important for common-sense reasoning and problem solving.

There are several directions for future work. First, it remains to identify optimization techniques that decrease the arity of the predicates in our rewritings in order to pave the way for efficient implementations. Of particular interest for implementation would be the rewriting to plain Datalog due to the importance of the target language. A challenging problem is to investigate whether our technique can be used to obtain a polynomial time Datalog rewriting for arbitrary CQs paired with Horn-$\mathcal {ALCHOI}$ or guarded TGDs, or to support other ontology or query languages, e.g., for $\mathcal {ALCHOI}$ with *number restrictions* and variations of *regular path queries* or for *warded* TGDs. A possible direction is to apply our method in contexts of other OMQ languages, e.g. for hybrid knowledge bases that combine DLs with Datalog rules, bringing some form of closed-word reasoning to DLs, or in extensions of OBDA with temporal constructs. It is known that such constructs often increase the complexity of OMQA and destroy FO-rewritability.
